# Preface: genomics and biology of exercise is undergoing a paradigm shift

**DOI:** 10.1186/s12864-017-4184-6

**Published:** 2017-11-14

**Authors:** Nir Eynon, Sarah Voisin, Alejandro Lucia, Guan Wang, Yannis Pitsiladis

**Affiliations:** 1 Institute of Sport, Exercise and Active Living (ISEAL), Victoria University, Melbourne, VIC Australia; 2 Murdoch Childrens Research Institute, Royal Children’s Hospital, Melbourne, Australia; 3 Universidad Europea de Madrid and Research Institute Hospital 12 de octubre, 28670 Villaviciosa de Odón, Madrid, Spain; 4 Centre of Sports Medicine for Anti-Doping Research, University of Brighton, Eastbourne, UK

Genomics and Biology of Exercise is a field of research aiming at understanding how genetic variations influence adaptations to exercise training in healthy and diseased populations, elite athletes, and predisposition to exercise-related injuries. While the pioneering HERITAGE family study (HEalth, RIsk factors, exercise Training And GEnetics) acknowledged the importance of large, collaborative enterprises to obtain reliable results [1], many of the studies in the field have suffered from methodological errors, resulting in invalidated and non-replicated results [2]. Consequently, after more than 20 years of research in the field there remains no set of genetic variants available to predict exercise performance and predisposition to injuries in individuals [3]. However, the field of Genomics and Biology of Exercise is undergoing an urgently needed paradigm shift as an ever increasing number of scientists realise that working together is the best way to advance the field.

## Why is working within consortia the best way to advance the field?

Consortia are initiatives that bring together efforts of several research groups, institutions and often several involved scientists from several countries to answer key questions in a field of research. There are many reasons why consortia are not only the preferred framework, but are actually necessary if we seek robust, reproducible, and translational results in exercise genomics.

It is now well-established that adaptations to exercise training, athletic performance, and predisposition to sports injuries are complex, polygenic traits that are highly heritable [2]. Heritability measures have been derived from twin and family aggregation studies [4, 5]; they correspond to the proportion of variance in performance and performance-related traits that is explained by heritable factors. Linkage analyses and genetic association studies allowed the identification of chromosomal regions and common single nucleotide polymorphisms associated with performance-related phenotypes [6]. However, the proportion of variance explained by all these variants has turned out to be much lower than the heritability measures derived from twin and family studies. This common phenomenon in complex traits is termed “missing heritability” [7] and it has been hypothesised that it occurs because traits are influenced by the combined influence of thousands of common genetic variants with very small effect sizes, as well as possibly rare variants with larger effect sizes [8, 9]. This directly implies that **large sample sizes** are necessary to obtain enough statistical power to detect those variants, and that **diverse cohorts** should be studied if we wish the whole population spectrum to benefit from research findings in exercise genomics. This is particularly the case for observational studies that require even larger sample sizes because of their uncontrolled setting [10]. Finally, **validation** and **replication** of findings is of paramount importance. For instance, the attempt to replicate genetic variants discovered in the HERITAGE Caucasian cohort showed very limited success [11]. This is where consortia can be helpful by raising the number of participants and providing a wide range of populations to study.

The current direction taken by exercise genomics strongly calls for the development of collaboration between research centres. It is becoming increasingly clear that we require to distance ourselves from small candidate-gene driven studies to obtain a more global, unbiased picture of how the genome influences the response to exercise by conducting whole genome approaches that are hypothesis-free such as **Genome-Wide Association Studies (GWAS)** [6]. GWAS study with elite athletes was published recently and was a fruit of the GAMES consortium [12]. The study involved a total of 1520 endurance athletes (835 who took part in endurance events in World Championships and/or Olympic Games) and 2760 matched sedentary controls. Given the low sample size, GAMES was underpowered to identify alleles with small effect sizes. However, some of the suggestive associations identified should be explored in expanded comparisons of world-class endurance athletes and non-athletic cohorts controls and in tightly controlled exercise training studies.

An observed association between genetic variants and phenotypes does not necessarily mean causation. Therefore, following the discovery and replication phase, **mechanistic and functional studies** are required to uncover how these variants act at the molecular level. Genetics is unlikely to yield robust results with clinical relevance unless it is combined with transcriptomics, proteomics and/or epigenetics to establish causal relationships between variants and phenotypes [13]. The **latest technological advances** such as next-generation sequencing and (epi)genome editing will also hopefully become routine procedures in the field of exercise genomics. A corollary is that **advanced bioinformatics and biostatistical analyses** to process and integrate such a heterogeneous, vast amount of data will be dearly needed. Here again, working in a consortium can help by bringing together different research groups that all specialise in different techniques that are complementary, and by splitting the amount of work to alleviate the burden put on each group. It should also be noted that from the fundamental biological ground work done at the genetic and molecular levels, we must then move to the applied field to improve clinical practice, standardised protocols to prevent injury, maximise training output and perhaps translate findings to different populations for treating muscle and cardiovascular-related diseases. Regular cross-talk between fundamental and applied research groups working in a consortium are needed to ensure that the exerted efforts at the fundamental level can be used and translated to a more applied and clinical setting later on.

## The establishment of the Athlome consortium and other consortia to speed up discoveries

Initiating large collaborations, especially between numerous research groups and institutions, are not an easy task. Each laboratory has its own standard operating procedures for essays and data storage/sharing, protocols and equipment. To obtain comparable results, collaborators require establishing common exercise protocols, standardising their testing procedures, updating some of their equipment, and establishing ground rules for data sharing without compromising their own independency. It is also important for all consortia members to explicitly voice their own goals and identities, to remap stakeholders and interests periodically and to adapt social and technical systems to emerging needs and practices [14]. Ultimately, all those initial efforts will be greatly rewarded by the significance and the relevance of the findings. It is virtually impossible for a single research group to conduct all the necessary studies that will answer questions in exercise genomics with sufficient depth. Consortia and large-scale studies are made to split the research effort and to speed up discoveries, thanks to each group’s expertise in different domains. For instance, the recently established **Athlome consortium project (** **www.athlomeconsortium.org** **)** is currently an umbrella for more than 15 research groups to share and combine genomic, epigenomic, transcriptomic, proteomic, and metabolomic data to address key questions in three main research areas: elite performance, training response, and predisposition to injuries [15]. Within the Athlome consortium, the **Gene SMART (Skeletal Muscle Response to Training)** study is an example of tightly controlled exercise training study collecting of a variety of muscle and blood phenotypes. This study is exploring ‘omics’ data to understand why some people are high responders while others are low responders to similar exercise training (The Methods paper is available in this issue). Another example is the National Institutes of Health Common Fund program titled Molecular Transducers of Physical Activity Consortium (MoTrPAC) that is gathering 23 institutions in a common effort to catalogue the biological molecules affected by exercise in people, to assemble a comprehensive map of the molecular changes that occur in response to movement and, when possible, relate these changes to the benefits of physical activity [3, 16]. These recently launched consortia demonstrate a strong drive to move the field of exercise genomics in to the next level, with great hope to see these consortia succeed where individual research groups have failed.

The manuscripts published in this issue summarise some of the recent research multi-centre advances in the field of exercise genomics. The overall goal, as stated elsewhere [15, 17, 18] is to identify the sequence variation that plays a causal role in response to exercise and athlete’s predisposition to injuries and illness, and then to use this information to generate insights into the biology of health and disease that can support clinical translation.
